# Using transcranial Direct Current Stimulation (tDCS) to investigate why faces are and are not special

**DOI:** 10.1038/s41598-021-83844-3

**Published:** 2021-02-23

**Authors:** Ciro Civile, Samantha Quaglia, Emika Waguri, Maddy Ward, Rossy McLaren, I. P. L. McLaren

**Affiliations:** School of Psychology, College of Life and Environmental Sciences, University of Exeter, Exeter, UK

**Keywords:** Psychology, Human behaviour, Cognitive neuroscience, Learning and memory

## Abstract

We believe we are now in a position to answer the question, "*Are faces special?*" inasmuch as this applies to the face inversion effect (better performance for upright vs inverted faces). Using a double-blind, between-subject design, in two experiments (n = 96) we applied a specific tDCS procedure targeting the Fp3 area while participants performed a matching-task with faces (Experiment 1a) or checkerboards from a familiar prototype-defined category (Experiment 1b). Anodal tDCS eliminated the checkerboard inversion effect reliably obtained in the sham group, but only reduced it for faces (although the reduction was significant). Thus, there is a component to the face inversion effect that we are not affecting with a tDCS procedure that can eliminate the checkerboard inversion effect. We suggest that the reduction reflects the loss of an *expertise-based* component in the face inversion effect, and the residual is due to a *face-specific* component of that effect.

## Introduction

We can rapidly recognize a face in a few milliseconds, and remarkably, after a glimpse, efficiently extract the information necessary to categorize a person’s facial expression, gender, ethnicity, and direction of gaze. This is a valuable ability, as accurate face recognition is crucial in social interaction, providing us with information about who we are interacting with and their motivational and emotional states. Understanding the nature of the mechanisms underpinning face recognition skills and whether these are face-specific or also applicable to other classes of stimuli is a fundamental question that researchers have been trying to answer for decades.

According to “*specificity*” accounts, neuro-cognitive mechanisms are selectively involved in processing faces per se and have little, if any, role in processing non-face stimuli^1^. Contrarily, an “*expertise*” account suggests that the neuro-cognitive mechanisms involved in face processing are elicited for all prototype-defined stimuli (i.e. that have a shared configuration) as long as we have been pre-exposed to them^2^. The argument between proponents of these two accounts has continued throughout the years and, if anything, increased with the advent of the cognitive neuroscience techniques that helped to inform this debate. Much of this evidence uses, as a robust behavioural index of face recognition skills, the face inversion effect^3–5^ which we introduce shortly, coupled with investigation of brain activation in the Face Fusiform Area (FFA) via fMRI or measurement of the event-related potential (ERP) component called the N170 using EEG.

We now believe that we are in a position to answer the question, "Are faces special?" Our answer is yes, that there is evidence for special processing that does not apply to other stimuli that upright faces benefit from, and that this contributes to the face inversion effect. But our answer is also no, that there is a component that is not specific to faces, that can be seen in other stimuli, and that is due to expertise resulting from experience with those stimuli. Our evidence for this answer is firstly, that we are able to show that experience with non-face stimuli (checkerboards) can produce an inversion effect. Secondly, our tDCS procedure can eliminate this inversion effect for these stimuli, but only partially reduces it for faces. Crucially, in these experiments we are able to show that this is the case using the same procedures and at the same level of performance for both types of stimulus. Our conclusion is that the significant reduction in the face inversion effect brought about by the tDCS reflects the loss of the expertise-based component of the face inversion effect, and that the residual face inversion effect is the other component that is not experience-based but rather due to specialised processing for faces. In what follows, we briefly review the current status of the debate, and then trace the development of our approach to this problem culminating in two experiments that provide evidence in favour of our answer.

*The Face Inversion Effect*: It can be defined as worse recognition performance to inverted compared to upright faces. When it was first discovered, the inversion effect was suggested to be a marker for the specificity account, because it is larger for faces than in response to other non-face stimuli^3,6^. This was later challenged by studies that showed that a large inversion effect could also be obtained with dog images or artificial stimuli (Greebles) or non-mono-orientated artificial stimuli such as checkerboards when participants either were or had become familiar with them^2,7,8^. This was impressive evidence for the expertise account of the inversion effect, but the specificity hypothesis received equally impressive support from work on the FFA.

*Imaging Studies*: The FFA is a cortical region in the fusiform gyrus that is found to be more highly activated when participants are presented with faces than when they viewed sets of non-face stimuli^9^. A face inversion effect (higher activation for upright vs inverted stimuli) was found at the FFA suggesting that a region specialised for processing faces was contributing to the inversion effect^10^. But importantly, an inversion effect on the FFA was also found for Greebles after (but not before) participants became familiar with them, which suggested that this region is not entirely specialized for faces. In recent years, some criticisms of this work have been raised by the same authors who first investigated the use of the inversion effect on the FFA as an index of face processing. The general issue that has emerged was that the inversion effect found for faces and particularly for Greebles at the FFA was relatively small, suggesting that fMRI may not be sensitive enough to pick up such effects reliably^11,12^. Moreover, fMRI has not produced anything like the robust face inversion effect reported on the ERP N170 component.

With more than 200 current publications, the N170 represents one of the most studied neuro-indices of face processing. It consists of a negative-polarity deflection maximal at 130 to 210 ms after the onset of a face stimulus, recorded at posterior temporal sites. Importantly, inverted faces elicit a *larger* (amplitude) and *delayed* (latency) N170 component compared to upright faces, this is what is commonly defined as the face inversion effect index on the N170^13,14^. It was considered consistent with the idea that faces are processed in a special way not applicable to other stimuli.

However, Busey and Vanderkolk^15^ showed that fingerprint experts exhibited a larger and delayed N170 for inverted fingerprints similar to that exhibited with inverted faces. Rossion et al.^16^ showed that after training the Greebles inversion effect on the N170 was comparable to that for faces. Furthermore, Civile et al.^17^ using an old/new recognition task, first established a behavioural inversion effect for prototype-defined categories of checkerboards (vs non-prototypical ones) after participants were pre-exposed to them. Following this, they showed a larger inversion effect on the N170 for checkerboards drawn from a familiar category than that for checkerboards drawn from a novel category. These results point towards an expertise-based account of the inversion effect on the N170.

Whilst much of the data reviewed so far supports an expertise account of the inversion effect on the N170, the key question about whether or not faces are special still has to be answered with regard to this measure. And, as in the case of studies using fMRI, results on the N170 component seem to provide some support for both the *specificity* and the *expertise* accounts. Some of the main issues related to the N170 component and the face inversion effect remain unsolved. Perhaps the biggest issue is the fact that often behavioural results are not simply related to the effects obtained on the N170, at least not in the way that one might expect based on an expertise account. For example, studies conducted using sets of configurally (spatial relationships among facial features) manipulated faces (e.g. scrambled or Thatcherised) showed that the behavioural reduction of the inversion effect was due to reduced performance for upright manipulated faces. However, on the N170 component, changes in the inversion effect were due mainly to the manipulated inverted faces either showing a reduced delay or increased or reduced amplitude compared to upright faces^18,19^.

*Neurostimulation*: A new line of research based on using a particular transcranial Direct Current Stimulation (tDCS) procedure has provided evidence that the inversion effect for checkerboards and that for faces share at least some of the same causal mechanisms. The tDCS apparatus consisted of a target channel electrode and a reference channel electrode both placed on the scalp and delivering continuous low electro-current stimulation typically between 1-2 mA. When the active anodal stimulation is delivered, the current induces depolarization of the resting membrane potential which increases neural excitability and allows for more spontaneous cell firing. Studies have shown that tDCS stimulation of a duration of 9–13 min generates after-affects that last for about 1 h after the end of the stimulation. The sham stimulation served as a control, and in this condition, tDCS is only delivered for a brief period of time (usually 30 s in total), not enough to induce any changes^20–23^.

In 2016, Civile et al.^24^ examined the effects of anodal tDCS delivered over the left dorsolateral prefrontal cortex (DLPFC) at prefrontal area Fp3 (10–20 EEG system) during the old/new recognition task used by Civile et al.^17^ to obtain the checkerboard inversion effect^24^. This specific brain region was targeted with tDCS because of a previous fMRI study showing increased brain activation during a categorization task involving two categories of prototype-defined checkerboards^25^. Using a double-blind, between-subjects design, the authors showed that anodal tDCS delivered at Fp3 site (for 10 min at 1.5 mA) eliminates the inversion effect repeatedly found in Civile et al.^17^ for familiar checkerboards index of perceptual learning manifesting as expertise^24^. This was due to a reduction in recognition performance for upright checkerboards compared to sham. Civile et al.^26^ extended the tDCS procedure to an old/new recognition task this time testing the face inversion effect. Experiments 1 and 2 produced a significant interaction between stimulus orientation (Upright / Inverted) and stimulation given (Anodal / Sham) reflecting the reduction of the inversion effect in the anodal group due to reduced recognition performance for upright faces. Experiment 3 (an *active control* study) confirmed that the same effects are not obtained by applying tDCS to a different target area. Whereas in Civile et al.^24^ the tDCS procedure completely eliminated the checkerboard inversion effect, in Civile et al.^26^ the face inversion effect was significantly reduced but still itself significant. Importantly, there are two main limitations to the results obtained in Civile et al.^24^. Firstly, overall performance was low, with recognition of both upright and inverted familiar checkerboards being numerically below chance level in some conditions. Secondly, the inversion effect for familiar checkerboards found in Civile et al.^17^ and Civile et al.’s^26^ sham group is a lot smaller than the traditional face inversion effect^24,26^.

Here we adopted the same tDCS procedure but now applied for the first time to a *matching task* involving faces (Experiment 1a) and checkerboards (Experiment 1b). This behavioural task is often used to test individuals with face-blindness, because it is easier to perform, ensuring a higher level of performance that otherwise would not be obtained using the old/new recognition task adopted to test the inversion effect^27,28^. As such, it should allow us to obtain a comparison of the inversion effect for faces and that for checkerboards by ensuring a high level of overall performance in both.

Our argument is as follows: if the tDCS procedure eliminates the inversion effect for checkerboards, but not for faces, this would provide direct behavioral evidence in support of two factors contributing to the effect; one based on expertise which we can fully influence with tDCS in the case of the checkerboards; the other partially based on a mechanism specific to faces which we cannot affect (overall face inversion effect would only be reduced compared to sham) with the same procedure.

## The study

### Method

#### Subjects

In total, 96 naïve (right-handed) subjects (30 male, 66 Female; Mean age = 20.9 years, age range = 18–27) took part in the two experiments. The sample size was decided based on previous studies that used the same stimuli, tDCS experimental procedure (double-blind, between subjects) and montage to modulate the inversion effect^18,26^. Subjects were students from the University of Exeter and were selected according to the safety screening criteria. All methods were performed in accordance with the relevant guidelines and regulations approved by the CLES Psychology Research Ethics Committee at the University of Exeter. We conducted a post-hoc power analysis (also Bayesian analyses reported in the results section) for our sample size in Experiment 1a using G*Power software (version 3.1.9.3), based on the effect size (η^2^_p_ = 0.15) recorded from the overall 2 × 2 interaction. This analysis revealed a statistical power of 0.99 (Effect size *f* = 0.42, 2 groups, 2 measurements). We conducted the same analysis for the sample size in Experiment 1b (η^2^_p_ = 0.13) which revealed a statistical power of 0.99 (Effect size *f* = 0.39, 2 groups, 2 measurements).

#### Materials

**Experiment 1a** used a set of 256 face images (5.63 cm × 7.84 cm, presented at a resolution of 1280 × 960 pixels) standardized to grayscale on a black background^18,26^. The original images were selected from the Psychological Image Collection at Stirling open database, (https://pics.stir.ac.uk). All the images were cropped to a standardized oval shape, removing distracting features such as the hairline, and adjusted to standardize image luminance. **Experiment 1b** used the checkerboard exemplars (5.50 cm × 5.50 cm, presented at resolution of 1280 × 960 pixels) created by Civile et al.^17^ (Experiment 1a). Category prototypes (16 × 16) were randomly generated with the constraint that they shared 50% of their squares with each of the other prototypes and were 50% black squares and 50% white. Exemplars were generated from these prototypes by randomly changing forty-eight squares thus, on average, 24 squares would be expected to alter from black to white or white to black^17^. Both Experiment 1a and 1b were run using Superlab 4.0.7b. on an iMac computer. Participants sat about 70 cm away from the screen on which the images were presented.

#### The tDCS paradigm

**Figure 1:**
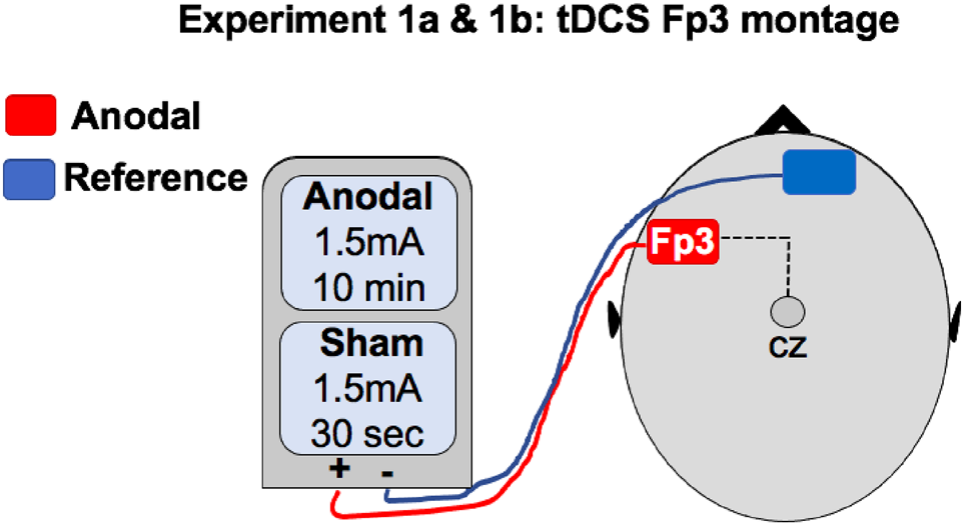
This illustrates the tDCS montage adopted in both Experiment 1a and 1b. This was the same tDCS montage adopted in Civile et al.^24,26^.

In both experiments we used the tDCS paradigm adopted in Civile et al.^24,26^. The stimulation was delivered by a battery driven constant current stimulator (neuroConn DC-Stimulator Plus) using a pair of surface sponge electrodes (7 cm x 5 cm i.e.35 cm^2^) soaked in saline solution and applied to the scalp at the target area of stimulation. A bilateral bipolar-non-balanced montage was used with one electrode (anode) placed over the target stimulation area (Fp3) and the other electrode (cathode) on the forehead over the reference area (right-eyebrow). The study was conducted using a double-blind procedure reliant on the neuroConn study mode in which the experimenter inputs numerical codes (provided by another experimenter unconnected with running the experiment), that switch the stimulation mode between “normal” (i.e. anodal) and “sham” stimulation. In the anodal condition, a direct current stimulation of 1.5 mA was delivered for 10 min (5 s fade-in and 5 s fade-out) starting as soon as the subjects began the first computer task and continuing throughout the study. Experiment 1a lasted approximately 13 min, whereas Experiment 1b lasted approximately 20 min. In the sham group, the identical stimulation mode was displayed on the stimulator and subjects experienced the same 5 s fade-in and 5 s fade-out, but with the stimulation intensity of 1.5 mA delivered for just 30 s, following which a small current pulse was delivered every 550 ms (0.1 mA over 15 ms) for the remainder of the 10 min to check impedance levels (Fig. 1).

#### The behavioural task

**Figure 2:**
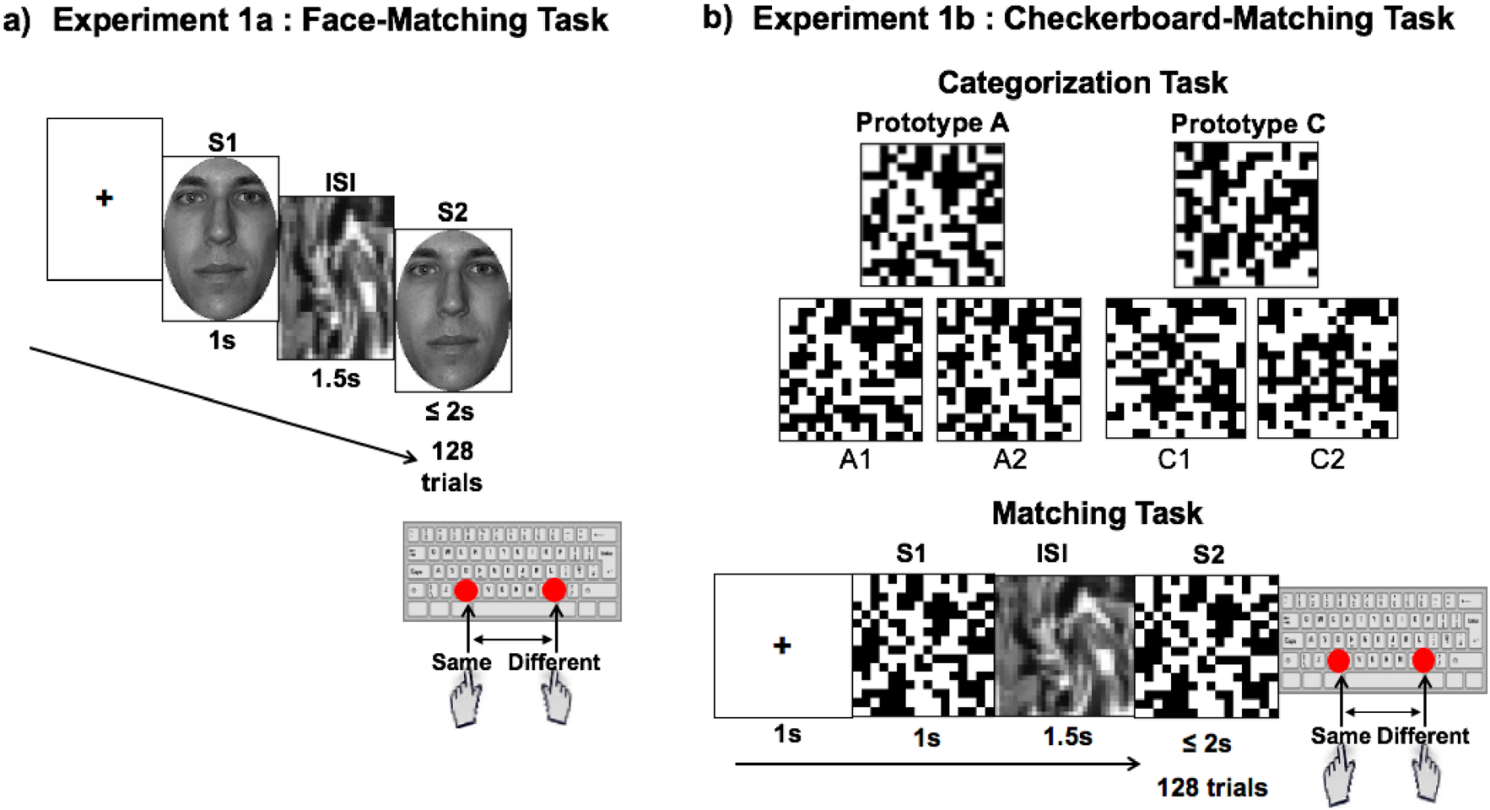
Panel (**a**) illustrates the behavioral task adopted in Experiment 1a. The original face images were selected from the Psychological Image Collection at Stirling open database, (http://pics.stir.ac.uk). Panel (**b**) illustrates the behavioral task adopted in Experiment 1b. The checkerboard exemplars were selected from Civile et al.^17^ (Experiment 1a).

In **Experiment 1a** once subjects gave their written informed consent, the instructions “*training phase*” were presented on the screen. The aim was for the subjects to associate the correct response keys ‘x’ or ‘.’ with the words SAME or DIFFERENT according to the allocated counterbalance condition. Overall, 48 trials (24 Same and 24 Different) were presented one at a time in random order for 1 s alternated with a fixation cue also presented for 1 s. Following this, subjects were presented with the instructions pertaining to the “face-matching” experimental task. Subjects were engaged in a *same/different* task over 128 trials. Each trial began with a fixation cue presented in the centre of the screen (1 s), followed by a TARGET face stimulus (1 s), an interstimulus interval (1.5 s) and a TEST face stimulus (≤ 2 s). Subjects pressed either the ‘x’ key or ‘.’ key to classify the test face as "same" or "different" to the target face. The first and second faces of a trial were always in the same orientation, and upright and inverted trials were randomly intermixed. The response keys were counterbalanced across subjects and corresponded to the same keys used in the training phase (Fig. 2, Panel a).

In **Experiment 1b** subjects were first engaged in a *categorization task* (pre-exposure/familiarization phase) where a set of checkerboards appeared on the screen, one at a time in a random order. Their task was to sort these exemplars into two categories (A & C) by pressing the two indicated keys. Subjects received immediate feedback as to whether their response was correct or incorrect. If no response was made within 4 s, they were timed out. The presentation of each checkerboard was signaled by a fixation cross in the center of the screen presented for 1 s. Overall 128 exemplars (64 drawn from category A and 64 from C) were presented. After the categorization task, subjects were engaged in the same training phase as that used in Experiment 1a. Following this, subjects performed a “checkerboard-matching” experimental task. Once again subjects were engaged in a *same/different* task over 128 trials. Each trial began with a fixation cue presented in the centre of the screen (1 s), followed by a TARGET checkerboard stimulus (1 s), an interstimulus interval (1.5 s) and a TEST checkerboard stimulus (≤ 2 s). Subjects pressed either the ‘x’ key or ‘.’ key to classify the test checkerboard as "same" or "different" to the checkerboard target. The first and second checkerboards of a trial were always in the same orientation, and upright and inverted (rotated by 180 degrees) trials were randomly intermixed. The response keys were counterbalanced across subjects and corresponded to the same keys used in the training phase. Half of checkerboards presented were new exemplars (not seen in the categorization task) drawn from category A, and the other half from category C (Fig. 2, Panel b).

#### Data analysis

In both experiments the primary measure was the accuracy data from all subjects in a given experimental condition which we used to compute a *d'* sensitivity measure for the matching task (based on performance on the same and different trials for each stimulus type) where a *d'* of 0 indicates chance-level performance. To calculate *d’*, we used subjects’ hit rate (H), the proportion of SAME trials to which the participant responded SAME, and false alarm rate (F), the proportion of DIFFERENT trials to which the participant responded SAME. There are two different methods of calculation that can be used in these circumstances that depend on the psychological model assumed for this task. One version, and perhaps the most commonly employed in this particular psychological context, uses the idea that the same / different judgement is made on the basis of a trace strength measure. The idea is that the target stimulus is encoded and sets up a trace that will then decay with time or be weakened by interfering intervening material. The test stimulus is then encountered, and an estimate of its trace strength made. If the test stimulus is the same as the target stimulus, then its trace strength should be high, if it is different, then it will be lower. In essence, this is the same sort of model that is used to explain old/new recognition in our earlier experiments and is also the model assumed in the original McLaren^8^ paper that used the current task, treating it as a variant of delayed matching. In this case, the method of calculating d' used by Stanislaw and Todorov^29^ is appropriate, as we simply have two distributions, one for matching stimuli and one for those that do not match, on the dimension of trace strength.

A somewhat different set of assumptions can be made however, in which the same / different judgement is more akin to deciding if two stimuli are identical on a psychophysical dimension such as brightness or loudness, and the method of computation for d' just referenced would not be appropriate in these circumstances. Now the model used assumes that when the test stimulus is encountered its deviation on this dimension from that of the target stimulus is assessed, and if the absolute magnitude of this deviation is large enough then the stimulus is classified as "different" with "same" being the outcome otherwise. In these circumstances, another method of calculating d' must be used, that due to Kaplan et al.^30^. This can give quite different values of d' for the same hit and false alarm rates, which raises the question of which is the right approach for this study, and whether the results we report would change depending on which model we used.

Our approach to resolving this issue in this paper is to calculate both forms of d'. We use the former method to enable comparison with earlier work in this area, but we have also repeated our analysis using the Kaplan et al.^30^ method as well. Apart from a change in the overall magnitude of d', we can confirm that the method used does not change the pattern of results and we report the alternative d' results in the supplementary material for this paper. In what follows, we give the d' based on the Stanislaw and Todorov^29^ method, each p-value reported for the comparisons between conditions is two-tailed, and we report the F or t value along with measures of effect size (Cohen's d or η^2^_p_) and for the critical interactions we also report the 95% confidence interval [CI] for Cohen's d as an aid to the interpretation of these effects.

To assess whether the results here obtained are commensurate with those obtained in Civile et al.^24,26^ studies using an old/new recognition paradigm, we also report Bayes factor analyses using the procedure outlined by Dienes^31^, assuming a one-tailed distribution for our theory and a mean of 0. We also provide additional analyses of this kind to help determine whether the results for the checkerboard stimuli can be genuinely taken as indicating a null effect for inversion rather than the reduced one observed with faces.

## Results

### Experiment 1a

**Figure 3:**
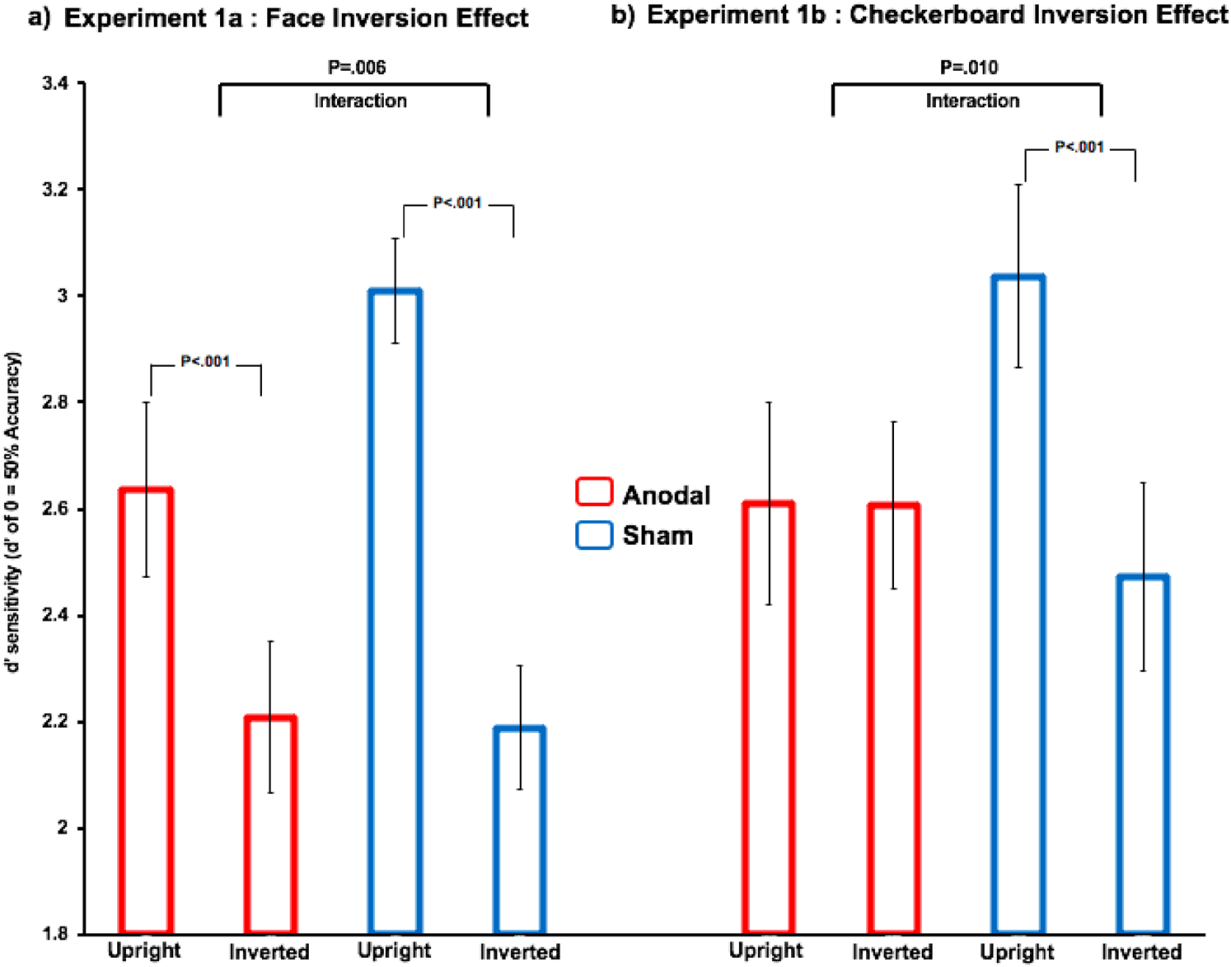
Panel (**a**) reports the results from Experiment 1a. Panel (**b**) reports the results from Experiment 1b. In both panels, the *x*-axis shows the stimulus conditions, the *y*-axis shows d'. Error bars represent s.e.m. In both experiments, performance against chance in both the sham and anodal groups was significantly above chance (for all conditions we found *p* < .001 for this analysis).

We computed a 2 × 2 mixed model design using, as a within-subjects factor, *Face Orientation* (upright or inverted), and the between-subjects factor *tDCS Stimulation* (sham or anodal). Analysis of Variance (ANOVA) revealed a significant main effect of *Face Orientation F*(1, 46) = 82.81, *p* < 0.001, η^2^_p_ = 0.64 indicating the standard inversion effect, and a significant two-way interaction, *F*(1, 46) = 8.17, *p* = 0.006, η^2^_p_ = 0.14, *d* = 0.82, CI = 1.44, 0.21, caused by the inversion effect being substantially reduced in the anodal group (Fig. 3, Panel a). As in Civile et al.^26^, no main effect of *tDCS Stimulation* was found confirming that the tDCS does not simply reduce overall performance, *F*(1, 46) = 1.06, *p* = 0.31, η^2^_p_ = 0.02. Follow-up paired *t* test analyses were conducted to compare performance on upright and inverted face stimuli (the inversion effect) in each tDCS group (sham, anodal). A significant inversion effect was found in the sham group (*M(difference)* = 0.822, *SD* = 0.52), *t*(23) = 7.67, *p* < 0.001, η^2^_p_ = 0.72, and a *reduced inversion effect* was found in the anodal group (*M(difference)* = 0.428, *SD* = 0.43), *t*(23) = 4.89, *p* < 0.001, η^2^_p_ = 0.51. We also compared the performance for upright faces in the two tDCS groups. This was done because, based on previous studies^26^ our tDCS procedure significantly affects upright faces but not inverted ones. An independent-sample t-test revealed a trend towards performance for upright faces in the anodal group (M = 2.63, SE = 0.80) being worse compared to that in the sham group (M = 3.01, SE = 0.48), *t*(46) = 1.95, *p* = 0.057, η^2^_p_ = 0.07. Finally, no significant difference was found between performance for inverted faces in the anodal group (M = 2.21, SE = 0.14) compared to that for inverted faces in the sham group (M = 2.19, SE = 0.12), *t*(46) = 0.105, *p* = 0.92, η^2^_p_ < 0.01.

### Experiment 1b

A 2 × 2 mixed model design using, as a within-subjects factor, *Checkerboard Orientation* (upright or inverted), and the between-subjects factor *tDCS Stimulation* (sham or anodal) revealed a significant main effect of *Checkerboard Orientation F*(1, 46) = 7.22, *p* = 0.010, η^2^_p_ = 0.14, reflecting the inversion effect, and a significant two-way interaction, *F*(1, 46) = 7.12, *p* = 0.010, η^2^_p_ = 0.13, *d* = 0.77, CI = 1.39, 0.15, which in this case signalled the absence of a significant inversion effect in the anodal group (Fig. 3, Panel b). No main effect of *tDCS Stimulation* was found, *F*(1, 46) = 0.429, *p* = 0.52, η^2^_p_ < 0.01. Follow-up paired *t* test analyses revealed a significant inversion effect in the sham group (*M(difference)* = 0.565, *SD* = 0.70) , *t*(23) = 3.94, *p* = 0.001, η^2^_p_ = 0.40, but this was not the case for the anodal group (*M(difference)* = 0.002, *SD* = 0.75), *t*(23) = 0.13, *p* = 0.99, η^2^_p_ < 0.01. We compared the performance for upright familiar checkerboards in the two tDCS groups as for Civile et al.’s^24^ study. Performance for upright familiar checkerboards in the anodal group (M = 2.61, SE = 0.93) was numerically reduced compared to that in the sham group (M = 3.03, SE = 0.84), *t*(46) = 1.66, *p* = 0.10, η^2^_p_ = 0.06. Finally, no significant difference was found between performance for inverted familiar checkerboards in the anodal group (M = 2.60, SE = 0.16) compared to that for inverted familiar checkerboards in the sham group (M = 2.47, SE = 0.18), *t*(46) = 0.575, *p* = 0.57, η^2^_p_ < 0.01.

### Analyses across experiments

We conducted an independent sample t-test using the inversion effect index (performance for upright – performance for inverted stimuli) for faces and checkerboards in the anodal groups which revealed a significant difference between these differences, *t*(46) = 2.40, *p* = 0.021, η^2^_p_ = 0.18, *d* = 0.69, CI = 1.30, 0.08. The same analysis for the inversion effect for faces and checkerboards in the sham groups did not give a significant difference on this measure, *t*(46) = 1.43, *p* = 0.158, η^2^_p_ = 0.11, *d* = 0.41, CI = 1.01, -0.18. Finally, we compared overall recognition performance across all the stimulus’ conditions averaged together in Experiment 1a (M = 2.51, SE = 0.10) vs Experiment 1b (M = 2.68, SE = 0.11), and we found no significant difference *t*(46) = 1.10, *p* = 0.28, η^2^_p_ = 0.02.

## Bayes factor analyses

### Experiment 1a

We conducted a Bayes analysis on the difference between the d’ values for upright and inverted faces (i.e. the inversion effect score) comparing the sham and anodal groups (thus capturing the 2 × 2 interaction) in Experiment 1a. We used as the *priors* the differences found in Civile et al.^26^ (Experiment 1 and 2 averaged together) setting the standard deviation of p (population value | theory) to the mean for the difference between the inversion effect in sham group vs that in the anodal group (0.30). We used the standard error (0.08) and mean difference (0.39) between the inversion effect in the sham group vs that in the anodal group in Experiment 1a. This gave a Bayes factor of 33,814, which is very strong evidence (greater than 10, for the conventional cut-offs see Jeffrey^32^) that these results are in line with our previous work i.e. the tDCS procedure used here reduces the face inversion effect.

Similarly, we also conducted a Bayes factor analysis using as priors the mean difference between sham upright faces and anodal upright faces found in Civile et al.’s^26^ Experiment 1 and 2 averaged together (0.28). We then used the standard error (0.11) and mean difference (0.37) between sham upright faces and anodal upright faces in Experiment 1a. This gave a Bayes factor of 98.35, which is also very strong evidence for the position that performance to upright faces is reduced by our tDCS procedure, consistent with our previous results.

### Experiment 1b

For Experiment 1b we conducted the same Bayes analyses as that for Experiment 1a but this time using as priors the means from the results obtained in Civile et al.^24^. We first took the differences found in Civile et al.^24^ (Experiment 1 and 2 averaged together) setting the standard deviation of p (population value | theory) to the mean for the difference between the inversion effect for familiar checkerboards in the sham group vs that in the anodal group (0.29). We used the standard error (0.14) and mean difference (0.57) between the inversion effect in the sham group vs that in the anodal group in Experiment 1b. This gave a Bayes factor of 570, which is very strong evidence that these results are in line with our previous work indicating that the tDCS reduces the inversion effect in checkerboards taken from a familiar, prototype-defined category.

We then calculated the Bayes factor using as priors the mean difference between sham and anodal upright familiar checkerboards found in Civile et al.’s^24^ Experiment 1 and 2 averaged together (0.31). We used the standard error (0.17) and mean difference (0.43) between sham and anodal upright familiar checkerboards in Experiment 1b. This gave a Bayes factor of 11.11, which is strong evidence that performance to upright familiar checkerboards is reduced by tDCS, and also consistent with our previous results.

Furthermore, we conducted a somewhat different Bayesian analysis for the effect of anodal tDCS on the checkerboard inversion effect in Experiment 1b. The question we tried to answer is the following: Given that the effect can be as large as is found in the Sham condition, is the effect in the Anodal condition part of that population, or is it better described as null (mean of zero)? We used as a prior the mean difference (upright – inverted) for the checkerboard inversion effect in the sham group (0.57), and the standard error (0.15) and mean difference (0) for the checkerboard inversion effect in the anodal group. This gave a Bayes factor of 0.25, which is less than 0.3. and hence can be considered as good evidence for the null. Moreover, we conducted the same calculations for Civile et al.’s^24^ Experiment 1 (Bayes factor = 0.53) and Experiment 2 (Bayes Factor = 0.31) resulting in an overall factor (across the three studies) of 0.04 which is strong evidence for the null, supporting the claim that our anodal stimulation eliminates the checkerboard inversion effect.

It could be argued, however, that whilst this convincingly establishes that the effect seen under anodal stimulation is not from the distribution that generated the sham condition results, it might still be generated by a distribution equivalent to that which produces the reduced (but not eliminated) inversion effect in the case of faces. In other words, the effect is not null, but rather drawn from a population with a reduced mean compared to sham. To assess this possibility, we conducted one final Bayesian analysis. This time we used not the raw mean difference for the sham group, but instead reduced it by the same fractional amount as was the case for the face results. Anodal stimulation reduced the inversion effect for the faces by a factor of 0.52 in Experiment 1a (mean anodal inversion effect = 0.43 / mean sham inversion effect = 0.82). We take this reduction as the typical reduction expected if the checkerboards are affected by our tDCS procedure in the same way as faces.

To reduce it by the same amount for the checkerboards, we use this factor and multiply it by the original effect found in the sham condition. Hence, this gave 0.52 × 0.57 = 0.30 for the checkerboard effect rather than the original 0.57 used in our previous calculation. Thus, we used 0.30 as the prior (the standard deviation of population value | theory), and the standard error (0.15) and mean difference (0) for the checkerboard inversion effect in the anodal group (Experiment 1b). This resulted in a Bayes factor of 0.45. This is less than 1, which is evidence for the null, but by no means conclusive evidence that our result could not be obtained from the "reduced" distribution used as a prior.

We then conducted a similar calculation for the checkerboard inversion effect studies in Civile et al.^24^. We first calculated the fractional reduction of the face inversion effect in the anodal vs sham in Civile et al.’s^26^ Experiment 1 (mean anodal inversion effect = 0.24 / mean sham inversion effect = 0.50) and Experiment 2 (mean anodal inversion effect = 0.027 / mean sham inversion effect = 0.62). This gave values of 0.48 for Experiment 1 and 0.43 for Experiment 2. We calculated the average of these values, 0.45, and multiplied it by the sham checkerboard inversion effect from Civile et al.’s^24^ Experiment 1 (0.27). This gave 0.45 × 0.27 = 0.12. Then we used 0.12 as the prior (the standard deviation of population value | theory), combined with the standard error (0.26) and mean difference (-0.12) for the checkerboard inversion effect in the anodal group from Civile et al.’s^24^ Experiment 1. This resulted in a Bayes factor of 0.78.

Finally, we multiplied 0.45 by the sham checkerboard inversion effect from Civile et al.’s^24^ Experiment 2 (0.18). This gave 0.45 × 0.18 = 0.08. Using 0.08 as the prior (the standard deviation of population value | theory), and the standard error (0.09) and mean difference (-0.05) for the checkerboard inversion effect in the anodal group from Civile et al.’s^24^ Experiment 2 resulted in a Bayes factor of 0.57. The overall Bayes factor for these three experiments (0.45 × 0.78 × 0.57) is now 0.20 which is good evidence for the null, supporting the claim that our anodal stimulation eliminates the checkerboard inversion effect.

## General discussion

In the two experiments here reported we investigated the effects of a particular tDCS procedure applied to the robust inversion effect typically found for faces (Experiment 1a) and that found for non-mono-orientated sets of stimuli (checkerboards) which participants have been familiarised with (Experiment 1b). In doing so, we aimed to provide the first direct evidence that a comparably robust inversion effect can be found with prototype-defined familiar categories of checkerboards to that for faces. We did this by adopting a face-matching task which ensured a high level of performance for the checkerboard stimuli as well as the faces. Importantly, in the sham group, the inversion effect for checkerboards and that for faces did not differ statistically. Given that we had achieved this, we could then investigate the extent to which our tDCS procedure could modulate the inversion effect for faces and for checkerboards. Here our results show that the tDCS procedure reduced the inversion effect for faces compared to sham (Experiment 1a) and the inversion effect for checkerboards compared to sham (Experiment 1b). Furthermore, in agreement with the previous results in the literature^24,26^ our Bayesian analyses also indicated that the reduction of the face inversion effect and the checkerboard inversion effect would both seem to rely mainly on the impairment in performance at recognising upright stimuli in the anodal group compared to sham.

So far there is a strong correspondence between the results using the two types of stimuli. Our critical finding from this study is that, whereas the anodal tDCS procedure eliminates the inversion effect for checkerboards whilst maintaining a high level of overall performance, the same anodal tDCS procedure significantly reduced inversion effect for faces (compared to sham) which however it still remains significant. Thus, when we directly compare the inversion effect for faces and that for the checkerboards in the anodal group, we find a significant difference. One possibility is that this finding is driven by the fact that there is a discrepancy in the level of performance for the two sets of stimuli (i.e. faces and checkerboards). The argument would be that our life-long expertise in seeing upright faces produces better performance to the faces that is robust to the impairment induced by the tDCS procedure and so the inversion effect is still significant. However, there are three main reasons to reject this argument. Firstly, a careful examination of the effects obtained shows that in the anodal group, recognition performance for upright faces and for upright checkerboards is at a similar level. Secondly, in the additional analyses across the experiments we directly compared the inversion effect in the sham group for faces vs the inversion effect in the sham group for checkerboards and we found no significant differences. Finally, in the additional analyses conducted across the experiments we found no significant differences between the overall recognition performance in Experiment 1a vs the overall recognition performance in Experiment 1b.

We can now interpret our main results in terms of the theory of perceptual learning at the heart of Civile, et al.^24,26^ studies, drawing what general conclusions we can from the work reported here and the work that led up to it. Importantly, Civile et al.^17^ suggested that the basis of the inversion effect for stimuli drawn from a familiar prototype-defined category (i.e. checkerboards) can be explained by the MKM model of perceptual learning. Specifically, the model predicts that it is elements that are relatively unpredicted by other elements present that will be salient, whereas those that are well predicted (by other elements of the stimulus) will be less salient. This follows from the salience modulation mechanism in the model which is a mechanism that gives rise to perceptual learning as a consequence of stimulus pre-exposure.

For example, in Experiment 1b, in the categorization task (i.e. the pre-exposure phase) participants learn how to categorize checkerboard exemplars drawn from two different categories. Each exemplar is constructed by adding noise to a prototype, and so each exemplar contains prototypical elements (i.e. features) that have not been changed as well as new elements that have. The former elements are those that the category prototype and any exemplars would tend to have in common. Due to the fact that these common elements are presented on most trials they would tend to lose their salience because of the associations that form between them. Consequently, these common elements become more predicted by associations because they are encountered nearly every time an exemplar is processed, and they are reliably predicted by many of the other elements present in the exemplar. Thus, when the categorization task ends these common elements would be strongly associated with the correct category, but they will now be relatively slow to form new associations because of the strong associations between them. This leads to perceptual learning, because the elements unique to each checkerboard exemplar will still have high salience due to their low exposure during the categorization task and the lack of other elements predicting them. And so, when participants are asked to discriminate between category exemplars (e.g. in a matching task or an old/new recognition task) it should be easier for them to do so considering that the salience of the elements that those exemplars share in common has now decreased, whereas the salience of the elements that distinguish them is still high. Critically, this advantage would be lost on inversion, because the model predicts that stimulus representations are orientation specific, and so participants are not familiar with the exemplars turned upside down; hence, the unique elements of an exemplar would no longer enjoy any salience advantage^33–36^.

Civile et al.^24,26^, interpreted the reduction of the inversion effect for checkerboards and faces by means of anodal tDCS, as being due to impaired recognition performance for upright stimuli based on the disruption of the salience modulation mechanism that would normally produce perceptual learning for upright stimuli. Specifically, when the anodal tDCS procedure is applied salience for the common elements between the category prototype and the exemplars if anything increases rather than decreases. The implication is that participants will now be better at learning about commonalities (i.e. the common elements) than differences (i.e. unique elements) between exemplars resulting in enhanced *generalisation*. This would have the consequence of making it harder to use the unique elements typical of each exemplar in order to discriminate it from other similar exemplars. Thus, the inversion effect seen with checkerboards and that for faces would be impacted by considerably reduced performance for upright stimuli. It is important to notice that the perceptual learning process as explained here applies mainly to upright familiar stimuli. This is because we have little or no experience in seeing, for example, faces presented upside down and so performance is not aided by any significant amount of perceptual learning for stimuli in this orientation.

These claims receive support from Experiment 1a and b in this paper, where we extended the effect of anodal tDCS at Fp3 on the inversion effect to a face-matching task. Our results contribute to the face recognition literature by providing a direct evidence for the reduction in the face inversion effect being partial and incomplete. Importantly, in the anodal group, the remaining face inversion effect was both significant in itself and significantly larger than the non-significant checkerboard inversion effect, suggesting that there is a component to the face inversion effect that is not due to expertise manifesting as perceptual learning and that we are not affecting it with our specific tDCS procedure which can eliminate the checkerboard inversion effect.

Future work should aim to investigate what specific component of the face inversion effect is not affected by our tDCS procedure. Interestingly, recent studies have shown that in addition to the specificity vs expertise main debate in face recognition, a third factor may be considered as well. Importantly, Zhao et al.^37^ showed that nonface stimuli (non mono-orientated line patterns) containing salient Gestalt information (i.e. connectedness, closure, and continuity between parts) can elicit face-like holistic/configural processing in the absence of expertise^37,38^. They used a composite face effect paradigm which involves presenting aligned and misaligned composite faces made by selecting the top and bottom halves from two separate faces and composite line patterns. The key finding was that participants showed a similar composite effect (better recognition of the top half of a face when conjoined with a *congruent*, in terms of the response required, rather than *incongruent* bottom half) for face and line patterns composite stimuli. Thus, the authors suggested that expertise is not always needed for specifically constructed nonface stimuli in order to elicit similar perceptual processes seen in face stimuli. We hypothesize that it may be holistic processes of this kind that are responsible for the residual face inversion effect in our study. Extending our tDCS procedure to the composite effect for faces and that for the kind of stimuli used in Zhao et al.^37^ may improve our understanding of the specific source of information influenced (or not) by our tDCS procedure. If we find that our procedure does not influence this type of composite effect whilst still affecting performance to upright faces, then this would support our hypothesis.

More generally, the results from Experiment 1a and 1b here reported also contribute indirectly to the emerging body of literature investigating the use of anodal tDCS delivered over the DLPFC to modulate various cognitive tasks. The DLPFC has often been suggested to be a source of top-down control that influences the course of bottom-up visual processing through increases in extra-striate neural activity by enhancing attention to elements of the visual field. However, the results of anodal tDCS applied to the DLPFC have sometimes been unclear (for a recent review see Tremblay et al.^39^). Ambrus et al.^40^ tested the effect of anodal tDCS delivered over the DLPFC at Fp3 during a categorization learning task testing the prototype distortion effect. This effect refers to the increased performance at categorizing category prototypes vs category exemplars, neither of which subjects had previously been trained on. The results from Ambrus et al.^40^ showed that anodal stimulation eliminated the prototype distortion effect by affecting participants’ ability to identify prototype and low distortion pattern exemplars as category members compared to sham (see also McLaren et al.^41^ and Kincses et al.^42^ for other studies that have used the same tDCS procedure on categorization learning tasks)^40–42^. In addition to categorization learning anodal stimulation over the left DLPFC was also shown to decrease working memory performance^43^, risk-taking behaviors^44^ negative emotion perception^45^ and cognitive flexibility^46^. However, other studies have shown anodal stimulation over the left DLPFC to increase working memory performance^47^ positive emotion processing^22^, performance on verbal tasks^48^ learning^49^ and mental flexibility including problem-solving, planning, and inhibition^50,51^. Taken all together, the results from these studies support the claim that tDCS at DLPFC can modulate numerous cognitive functions, but to properly interpret the results, a clear a priori hypothesis based on a theoretical background is necessary if we are to be able to interpret the results. Also, careful technical (e.g. stimulation intensity and duration) and methodological considerations (e.g. double-blind procedure) are mandatory to obtain further insights into the impact of tDCS on cognitive functions and related behavioural effects.

Coming back to the question concerning whether faces are special, our findings are congruent with the idea that two factors are contributing to face recognition skills. On this account, our tDCS procedure removes the expertise component in both faces and checkerboards by impairing recognition for upright stimuli, and in doing so eliminates the checkerboard inversion effect (because this was entirely dependent on familiarity with the category) but only reduces that for faces because it is not entirely expertise-based. The remaining inversion effect, we suggest, may involve mechanisms that are face-specific. Our conclusion is that the evidence we present here is consistent with the position that faces both are, and are not, special.

## Supplementary Information

Supplementary Information
