# Supplementary material for: Using transcranial Direct Current Stimulation (tDCS) to investigate why faces are and are not special
Source: Sci Rep. 2021 Feb 23;11:4380. doi: 10.1038/s41598-021-83844-3 (PMC7902624; doi:10.1038/s41598-021-83844-3)
Supplement: Supplementary file 1 — Supplementary Information [file 41598_2021_83844_MOESM1_ESM.docx]

**Supplemental Material for:**

**Using transcranial Direct Current Stimulation (tDCS) to investigate why Faces are and are Not Special**

Ciro Civile, Samantha Quaglia, Emika Waguri, Maddy Ward, Rossy McLaren, and I.P.L. McLaren

**Results based Kaplan et al (1978)’s method of calculating d-prime**

***Experiment 1a***

We computed a 2 x 2 mixed model design using, as a within-subjects factor, *Face Orientation* (upright or inverted), and the between-subjects factor *tDCS Stimulation* (sham or anodal). Analysis of Variance (ANOVA) revealed a significant main effect of *Face* *Orientation F*(1, 46) = 91.65, *p* < .001, η^2^_p_ = .66 indicating the standard inversion effect, and a significant two-way interaction, *F*(1, 46) = 8.77, *p* = .005, η^2^_p_ = .16, *d* = 0.85, CI = 1.47, 0.23, caused by the inversion effect being substantially reduced in the anodal group (Figure S1, Panel a). As in Civile, McLaren and McLaren (2018)^26^, no main effect of *tDCS Stimulation* was found confirming that the tDCS does not simply reduce overall performance, *F*(1, 46) = .932, *p* = .33, η^2^_p_ = .02. Follow-up paired *t* test analyses were conducted to compare performance on upright and inverted face stimuli (the inversion effect) in each tDCS group (sham, anodal). A significant inversion effect was found in the sham group (*M(difference)* = .1.04, *SD* = .61), *t*(23) = 8.35, *p* < .001, η^2^_p_ = .75, and a *reduced* *inversion effect* was found in the anodal group (*M(difference)* = .549, *SD* = .53), *t*(23) = 5.01, *p* < .001, η^2^_p_ = .52. We also compared the performance for upright faces in the two tDCS groups. This was done because, based on previous studies^26^ the tDCS procedure significantly affects upright faces and not inverted ones. An independent-sample t-test revealed a trend towards performance for upright faces in the anodal group (M= 4.20, SE=.20) being worse compared to that in the sham group (M= 4.66, SE=.11), *t*(46) = 1.94, *p* = .058, η^2^_p_ = .07. Finally, no significant difference was found between performance for inverted faces in the anodal group (M= 3.66, SE=.18) compared to that for inverted faces in the sham group (M= 3.62, SE=.15), *t*(46) = .151, *p* = .88, η^2^_p_ < .01.

***Experiment 1b***

A 2 x 2 mixed model design using, as a within-subjects factor, *Checkerboard Orientation* (upright or inverted), and the between-subjects factor *tDCS Stimulation* (sham or anodal) revealed a significant main effect of *Checkerboard* *Orientation F*(1, 46) = 7.64, *p* = .008, η^2^_p_ = .14, reflecting the inversion effect, and a significant two-way interaction, *F*(1, 46) = 7.10, *p* = .011, η^2^_p_ = .13, *d* = 0.77, CI = 1.38, 0.15, which in this case signaled the absence of a significant inversion effect in the anodal group (Figure S1, Panel b). No main effect of *tDCS Stimulation* was found, *F*(1, 46) = .731, *p* = .40, η^2^_p_ = .01. Follow-up paired *t* test analyses revealed a significant inversion effect in the sham group (*M(difference)* = .745, *SD* = .91) , *t*(23) = 3.98, *p* = .001, η^2^_p_ = .41, but this was not the case for the anodal group (*M(difference)* = .013, *SD* = .98), *t*(23) = .069, *p* = .95, η^2^_p_ < .01. We compared the performance for upright familiar checkerboards in the two tDCS groups as for Civile et al (2016)’s study^24^. Performance for upright familiar checkerboards in the anodal group (M= 4.19, SE=.20) was numerically reduced compared to that in the sham group (M= 4.79, SE=.23), *t*(46) = 1.93, *p* = .06, η^2^_p_ = .06. Finally, no significant difference was found between performance for inverted familiar checkerboards in the anodal group (M= 4.17, SE=.19) compared to that for inverted familiar checkerboards in the sham group (M= 4.04, SE=.22), *t*(46) = .434, *p* = .66, η^2^_p_ < .01.

***Analyses between experiments***

We conducted an independent sample t-test using the inversion effect index (performance for upright – performance for inverted stimuli) for faces and checkerboards in the anodal groups which revealed a significant difference between these differences, *t*(46) = 2.34, *p* = .023, η^2^_p_ = .11, *d* = 0.68, CI = 1.28, 0.06. The same analysis for the inversion effect for faces and checkerboards in the sham groups did not give a significant difference on this measure, *t*(46) = 1.31, *p* = .193, η^2^_p_ = .07. Finally, we compared overall recognition performance across all the stimulus’ conditions averaged together in Experiment 1a (M= 4.04, SE=.13) vs Experiment 1b (M= 4.30, SE=.15), and we found no significant difference *t*(46) = 1.33, *p* = .20, η^2^_p_ = .03, *d* = 0.38, CI = 0.98, -0.21


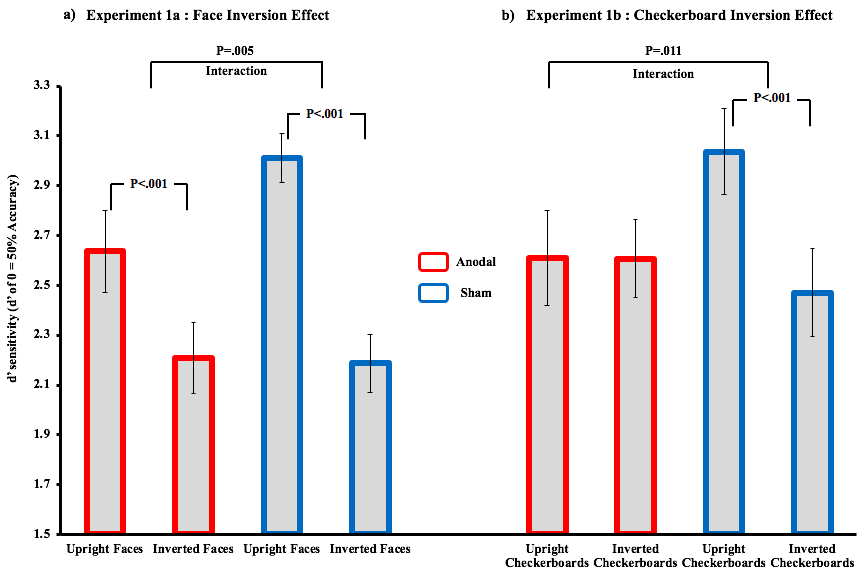


**Figure S1. Panel a** reports the results from Experiment 1a. **Panel b** reports the results from Experiment 1b. The *x*-axis in both panels shows the stimulus conditions, the *y*-axis shows d'. Error bars represent s.e.m. In both experiments, performance against chance in both the sham and anodal groups were significantly above chance (for all conditions we found *p* < .001 for this analysis).
